# Case for diagnosis. Pruritic erythematosquamous lesion in the auricle^☆^^☆☆^

**DOI:** 10.1016/j.abd.2019.11.011

**Published:** 2020-05-11

**Authors:** Elaine Dias Melo, Patrícia Motta de Morais, Débora Cristina de Lima Fernandes, Paula Frassinetti Bessa Rebello

**Affiliations:** a Teaching and Research Department, Fundação de Dermatologia Tropical e Venereologia Alfredo da Matta, Manaus, AM, Brazil; b Department of Histopathology, Fundação de Dermatologia Tropical e Venereologia Alfredo da Matta, Manaus, AM, Brazil; c Laboratory of Mycology, Fundação de Dermatologia Tropical e Venereologia Alfredo da Matta, Manaus, AM, Brazil; d Department of Tropical Dermatology, Fundação de Dermatologia Tropical e Venereologia Alfredo da Matta, Manaus, AM, Brazil

**Keywords:** Auricle, Chromoblastomycosis, Ear, external, Mycosis

## Abstract

Chromoblastomycosis is a subcutaneous mycosis with chronic evolution that mainly affects the lower limbs and, less frequently, the auricles. Clinically, it presents with papillary verrucous, nodular, and/or tumoral lesions, whether isolated or infiltrated, forming plaques and, sometimes, atrophic in some areas. Histopathologically, it is characterized by a dermal granulomatous inflammatory infiltrate, and the diagnosis can be confirmed by the presence of fumagoid bodies in anatomopathological or direct mycological exams. The treatment to be indicated will depend on the extent and location of the lesions, using systemic antifungals, surgical removal, cryotherapy, thermotherapy, and immunoadjuvants. The present study reports an atypical presentation of chromoblastomycosis on the auricle.

## Case report

**Figure 1:**
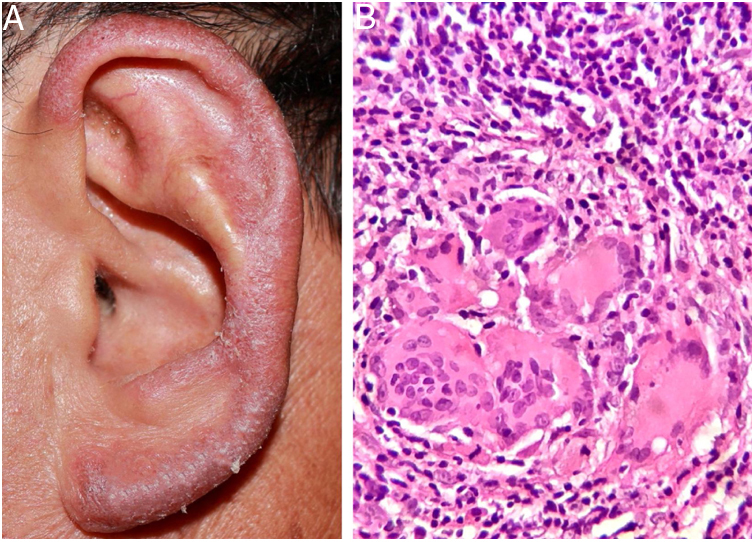
(A and B) Erythematous, infiltrative lesion in the left ear. Histopathological examination: granulomatous inflammatory infiltrate with suppurative foci (Hematoxylin & eosin, ×400).

A 57-year-old male farmer presented a pruritic lesion on the left auricle, with 20 years of evolution. He did not recall local trauma at the onset of the condition. Upon examination, an infiltrated, erythematosquamous lesion was observed on the left ear. The histopathological examination presented a granulomatous inflammatory infiltrate with suppurative foci (Fig. 1A and B).

What is your diagnosis?

1. Jorge Lobo's disease
2. Sarcoidosis
3. Paracoccidioidomycosis
4. Chromoblastomycosis

The review of the sections for histopathological examination evidenced the presence of fumagoid bodies, compatible with chromoblastomycosis.

**Figure 2:**
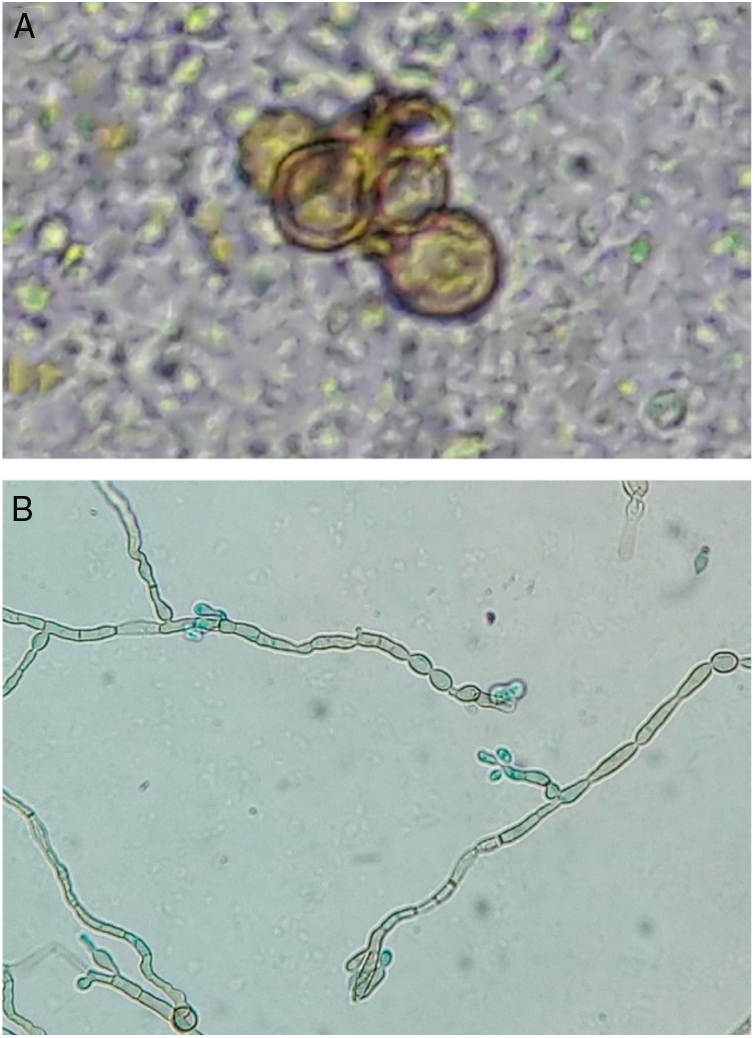
(A and B) Direct examination: fumagoid bodies. Microculture: septate demaceous hyphae and elliptical conidia in the upper portion of simple or slightly branched conidiophores – phenotypic characteristics compatible with *Rhinocladiella* spp. (KOH-20%, ×400; blue, lactophenol, ×400).

In the direct examination of biopsy fragments in 20% KOH, septate demaceous hyphae and isolated yeast cells in pairs were observed, as well as some gemmule structures. In culture, on Mycosel agar (DIFCO®) and Sabouraud dextrose agar (DIFCO®) with chloramphenicol (0.05 g/L), a blackened colony growth was observed; in microculture, phenotypic findings compatible with *Rhinocladiella* *sp.* were observed (Fig. 2 A and B).

*R. aquaspersa* was confirmed through amplification and sequencing of the intergenic spacer (ITS) region of the ribosomal DNA (rDNA) using the polymerase chain reaction (PCR) technique.

**Figure 3:**
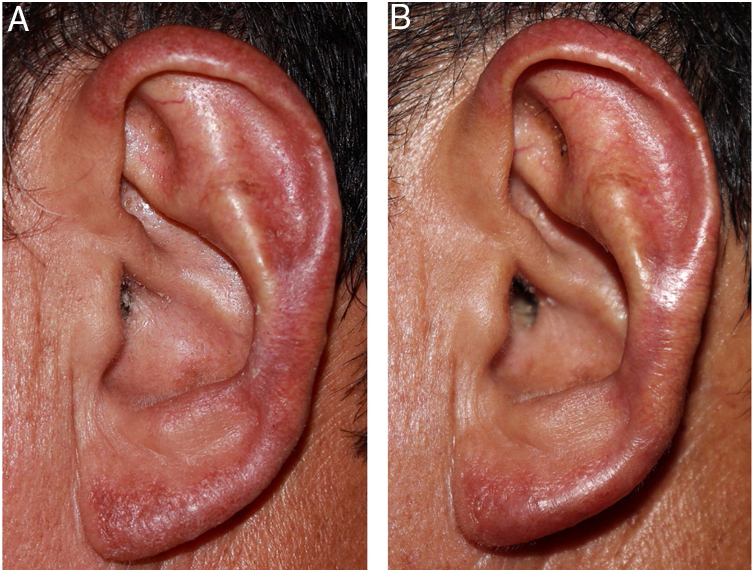
(A and B) After 25 days of treatment, and at the end of ten weeks.

After confirmation of the diagnosis of chromoblastomycosis, itraconazole (300 mg/day) was initiated; the patient presented significant improvement after 25 days and almost complete remission in ten weeks (Fig. 3 A and B). The patient remains under outpatient follow-up.

## Discussion

Chromoblastomycosis is a subcutaneous mycosis caused by demaceous fungi of the order Chaetothyriales, family Herpotrichiellaceae, found in decomposing soil, vegetables, plants, and wood.^1^, ^2^ The main etiological agents are from the genera *Fonsecaea*, *Cladophialophora*, *Phialophora*, *Rhinocladiella,* and *Exophiala*. Inoculation occurs after trauma with contaminated material.^1^, ^3^, ^4^

The condition most frequently affects men between 40 and 50 years of age, being considered a cosmopolitan disease, with greater prevalence in tropical and subtropical regions.^2^ In Brazil, it occurs in most states, with a predominance in the Amazon region, particularly in the state of Pará.^2^, ^5^

The disease mainly affects the lower limbs. In cases with long evolution, association with lymphedema is common.^1^, ^4^, ^6^ In Japan, the most common locations involved are the upper limbs, face, and cervical region.^2^ Reports of manifestations exclusively on the auricle are rare.^1^, ^3^, ^6^, ^7^, ^8^, ^9^, ^10^ Among the cases reported in this topography, *Fonsecaea* *pedrosoi*^1^, ^3^, ^10^ and *Phialophora* *verrucosa*^7^ were the most commonly identified agents, followed by *Fonsecaea* *nubica*^6^ and *Rhinocladiella aquaspersa*.^8^

According to the literature consulted, this is the second case with isolated involvement of the auricle caused by *R. aquaspersa*.

In the Amazon region, the differential diagnosis must include Jorge Lobo's disease, leprosy, anergic leishmaniasis, cutaneous tuberculosis, and paracoccidioidomycosis. Histopathological and mycological exams are essential for diagnosis.

Several treatments are indicated for chromoblastomycosis. For localized lesions, surgical excision, cryotherapy, or thermotherapy are recommended; for more extensive cases, systemic and immunoadjuvant antifungals are recommended.^2^, ^4^ Among systemic antifungals, itraconazole, posaconazole, voriconazole, and isavuconazole are used.^4^ The literature reports two cases of chromoblastomycosis in auricular locations, which presented complete regression after treatment with flucytosine^3^ and itraconazole^1^ for 12 and 10 weeks, respectively.

There are reports of a good therapeutic response with the association of systemic antifungals and topical immunoadjuvants, such as imiquimod.^4^

## Financial support

None declared.

## Authors’ contributions

Elaine Dias Melo: Conception and planning of the study; elaboration and writing of the manuscript; obtaining, analyzing, and interpreting the data; intellectual participation in propaedeutic and/or therapeutic conduct of studied cases; critical review of the literature.

Patrícia Motta de Morais: Approval of the final version of the manuscript; intellectual participation in propaedeutic and/or therapeutic conduct of studied cases; critical review of the literature; critical review of the manuscript.

Débora Cristina de Lima Fernandes: Elaboration and writing of the manuscript; critical review of the literature.

Paula Frassinetti Bessa Rebello: Approval of the final version of the manuscript; intellectual participation in propaedeutic and/or therapeutic conduct of studied cases.

## Conflicts of interest

None declared.
